# Hospital and Institutional News

**Published:** 1920-01-10

**Authors:** 


### HOSPITAL AND INSTITUTIONAL NEWS.

### A MEDICAL PEERAGE.

THE KING has been pleased to signify his intention of conferring a peerage of the United Kingdom on Sir Bertrand Edward Dawson, G.C.V.O., C.B., M.D., F.R.C.P., Physician to the London Hospital. Such an honour will be accepted by the whole profession of medicine as a recognition of the fine work done by medical and scientific men during the war period. The peerage is, indeed, an event in medical history, in that it is the first honour of the kind conferred upon a physician in actual practice. Lister's title was a national seal set upon his international fame, and Lord Ilkeston, who will be remembered as Sir Walter Foster, earned the honour by his political work. It may be said that in both instances the possession of a medical degree was an incident having relatively little to do with the award. Sir Bertrand Dawson, on the other hand, is a leading consulting physician and owes his peerage to all which underlies that position. Consulting Physician to the British Army during the war, Dean of the Faculty of Medicine, and a member of the Senate in London University, he holds rank in his profession worthy of his skill as a teacher and clinician. He is Physician-in-Ordinary to the King and also chairman of the Medical Consultative Committee instituted under the Ministry of Health. Already many of the distinctions which his medical brethren could confer have been showered upon him, and now his fellow practitioners will again congratulate him and welcome the new recognition given to medicine in his person.

### MEDICAL DISCOVERY.

IT is a little surprising that more attention has not been given in the medical journals to a matter upon which the recent letters in the *Times* from Sir Ronald Ross have touched at one vital point at least. The whole idea is *encouragement* of medical research, or, more strictly, medical discovery. The suggestions put forward involve a sum of only £15,000 annually, to be distributed in life pensions among men who have actually benefited the Empire by important medical discoveries. As the writer points out, this amounts to only one-thousandth part of the sum proposed to be allotted to aeronautics, one-twentieth that which Parliament grants to its own members or provides for political and legal work, and one-quarter what it gives as a subsidy for current medical investigations, and far less than the incomes of many wealthy individuals! Most of the comments we have seen and heard imply that the sum cannot be asked for at a time like the present. Yet what is the annual cost in sickness to the country? What standard of physical fitness and percentage of preventable disease did the recent national mobilisation bring to light? The suggestion is excellent in spirit and design, and yet it is held as impracticable in many quarters. It is almost unbelievable that a Government with such great and extensively advertised plans for the reconstruction of our national health should not realise its inevitable indebtedness when once this medical success is achieved. To the full do we agree with Sir Ronald Ross when he says: "My whole plea is that the world would be wiser to pay that little debt as a premier obligation."

### PENSIONS ORGANISATION.

IN a recent issue of the *Times* we read a concise description of the most recent hospital and general developments which have taken place under the Ministry of Pensions' control, and also an appreciation of the value and completeness of these measures. We recall the bitterness of our contemporary against the weakness of any early assistance and care given to our disabled men and the then general inadequacy of Government provisions. At the time these criticisms were in a large measure justified, but there is no doubt that very great improvement indeed has now been made. It is a pleasure to see the readiness with which it is now recorded that the changes made by Colonel W[illegible] the Director-General, go further in the right direction than was suggested, and that at the present moment the pensioner is among the most carefully examined and treated patients in the land.

### AUSTRALIAN SECRETARIAL APPOINTMENTS.

AT a meeting of the managers of the Alfred Hospital held in Melbourne Captain Glen was appointed secretary and superintendent in succession to Mr. R. Chambers Norman, resigned. Captain Glen has held several administrative positions in some of the largest A.I.F. hospitals in Egypt, France, and England, and has recently been secretary to a special influenza hospital near Melbourne. Mr. R. H. Metcalfe, chief clerk, who has been acting secretary and superintendent for the last four months, was officially appointed assistant secretary and superintendent. The retired administrative head, Mr. Norman, had filled the position of secretary and superintendent for over thirty-one years, and prior to his appointment was secretary of the Adelaide Children's Hospital for over five years, and of the Austin Hospital for Incurables, Melbourne, for fifteen months: an aggregate hospital service of thirty-eight years.

### NATIONAL HOSPITAL POST-GRADUATE COURSE.

THE Medical School of the National Hospital for the Paralysed and Epileptic, Queen Square, is following up the two successful courses of neurological instruction given in 1919 by a third course which will commence on the 19th inst. A conveniently arranged syllabus can be obtained on application to the Dean. The fee for the general course of lectures and clinics is £7 7s., and we notice that it is proposed to arrange a concurrent course in practical pathology for which the special fee of £5 5s. is required. The National Hospital should be in an excellent position to offer facilities to American and other graduate students who formerly proceeded to the clinics of Vienna and Berlin. The present thoroughly complete course should go a long way to emphasise the excellence of the English schools.

### THE SHERINGHAM DAYLIGHT.

This invention, a demonstration of which was held at the Imperial Institute of Science and Technology, South Kensington, on January 7 and 8 from 2 to 9 P.M., is a remarkably good reflector for correcting artificial light, the result approximating closely to daylight. The true balance of light is immediately apparent when examining coloured objects under the corrected rays. Even the most delicate variations of blue, yellow, and violet, which ordinarily cannot properly be distinguished under artificial light, became as clearly differentiated as by light from a north window. One has to think but a moment to realise the great possible value of this development to industry, art, and science. Printing of every kind, municipal lighting, art galleries come to mind among a host of other directions in which the invention may be employed. The originator is Mr. George Sheringham, who has received assistance in the final perfection from Mr. L. C. Martin, D.I.C., A.R.C.S., B.Sc., lecturer on optical engineering at the Imperial College of Science, and from Major Adrian Klein, M.B.E., late director of experimental work at the Camouflage School.

### A GUINEA-PIG SHORTAGE.

In view of the difficulty experienced in obtaining these animals for laboratory purposes in this country and to illustrate one of the many reasons for the great increase in price of medical biological products, we draw attention to the reported situation at so famous a centre as the Pasteur Institute in Paris. Before the war the breeding was carried on by people, chiefly women, of the countryside, but since 1914, when even the women had no time to devote to them, the scarcity has increased daily. The price has indeed been well over two francs each, and at the moment even the Pasteur Institute is unable to continue its experiments into the effects of serums against infectious disease.

### SIR ASTLEY COOPER'S LIFE.

In 1806 Mr. Astley Cooper left St. Mary Axe and moved to a house in New Broad Street, where during the next ten years he led an extremely busy life, and carried on a most remunerative private practice. At this time his day's work commenced as early as six o'clock and he was wont to carry out private dissections until eight, the next half hour being devoted to breakfast: subsequently his rooms were crowded with patients until one o'clock, when he would leave for Guy's Hospital. At the hospital, crowds of students waited for him, and he speedily commenced his teaching round the surgical cases. Shortly after he lectured on anatomy in Old St. Thomas's Hospital for an hour or more, and then visited the dissecting-room. Between five and six o'clock he usually visited private patients or else performed operations, finally reaching home about 6.30 P.M., but even in his carriage this tireless man would dictate notes to his assistants, or else discourse on the events of the day. After dinner he had a habit of sleeping for about ten minutes, and then if it were a lecture night, would start out again to give his surgical lecture! At this time his income exceeded the sum of £21,000 in a single year, and he had reached his zenith of prosperity.

### A CONTRAST IN SALARIES.

Two advertisements from the same column of the *Times*, of January 5, offer an extraordinary contrast in the salaries announced for two vacant posts, one of which is of rather an unusual type. To begin with, the Cancer Hospital offers £750 "inclusive" (which may be supposed to mean "without additional war bonus") for a successor to the late Mr. Howell, whose lamented death was recently announced in these pages (The Hospital, January 3, 1920, p. 299). On this no comment is necessary, except that the managers have evidently decided to have a good man and will probably get several to choose from. The other is an advertisement inserted by the House Governor of the General Hospital, Birmingham, for a "Food Supervisor," who is "to take entire charge of and be responsible for the purchase, preparation. and distribution of food" for staff and patients, numbering about 600. The salary offered is £130, with board, residence. and uniform. We do not hesitate to say that at the present time the General Hospital, Birmingham, does not deserve to get a satisfactory official for this paltry salary. If competition is such that suitable candidates appear, we shall still hold that to underpay an officer entrusted with duties so important and exposed to temptations, such as must accompany those duties, is false economy and a bad example. On the other hand, the idea of specialising the food department under the control of an individual appears to be excellent.

### ANGLICAN WAR MEMORIAL, BRISBANE.

The ceremony of laying the foundation-stone of a new hospital, to be called "St. Martin's War Memorial Hospital," has been performed by His Excellency the State Governor, Sir Hamilton Goold-Adams. St. Martin's Hospital covers portions of the Cathedral grounds. The architectural design of the hospital will be made to harmonise with the other Cathedral buildings. When completed the hospital is expected to be one of the finest of its class in Australia. The planning of the institution has been worked out on the "centralisation and radiation" principle. Accommodation for between fifty and sixty patients will be provided, principally in single bedrooms, but a few two- and four-bed wards will be built. The cost of the building is estimated at £5,000, of which rather more than half has been subscribed.

### A HOSPITAL MANAGER'S MUNIFICENT BEQUESTS.

Numerous generous bequests to London and local institutions are contained in the will of the late Mr. George Tempest Wade, J.P., of Leicester. Mr. Wade was for many years a member of the

Board of Governors of the Leicester Royal Infirmary, and was widely known as the Secretary of the Quorn Hunt, and as a local auctioneer and judge of horses. To the Leicester Royal Infirmary he bequeaths £1,500, subject to duty, to endow a bed; £1,500 to the Children's Hospital associated with the Royal Infirmary to endow a cot or cots; £1,000 to St. Luke's Home for the Dying, London; £1,000 to the Cancer Hospital, London; £1,000 to the Charnwood Convalescent Home for Children (Woodhouse Eaves, Leicestershire); £1,000 to the Royal Society for the Prevention of Cruelty to Children; £500 to the Blind Institution (Leicester); £500 to the Royal Hospital for Incurables, Leamington; £500 to the Royal Hospital for Incurables, Putney; £300 to the Royal Society for the Prevention of Cruelty to Animals; £300 to the Naples Society for the Protection of Animals; £200 to the Leicester Home for Waifs and Strays; £100 to Borstal Parish Church for the erection of a memorial window to the memory of his late wife; and numerous bequests to relations, partners, employees, and friends. The residue of his estate is bequeathed to his daughter for life and then to her children. The gross value of the estate is proved at £139,355. The total bequests to charities amount to £9,300.

### A HOSPITAL BUILT ON AN HISTORIC ABBEY.

From particulars recently published by the Sub-Warden and Bursar of St. Augustine's College, Canterbury, of the excavations which have been carried out to reveal the remains of St. Augustine's Abbey Church, it is apparent that some of the ground which has still to be investigated is encumbered by the property of a "hospital committee," presumably that of the Kent and Canterbury Hospital. "As soon as the hospital committee have cleared the ground," says the Bursar, "the further excavations can proceed without delay"—provided the requisite funds, for which he is appealing, are subscribed. The work already done has revealed the outline of the whole of the Norman church, as well as the original porticus of St. Martin's, completed in 613. The hospital works referred to include the building of a new laundry and the removal of the mortuary, and are expected to be completed within a few months. Wishing every success to the excavators in their task of uncovering monuments of antiquity, and approving fully of the accommodating spirit which the hospital committee displays, it is also possible to feel that this sacred ground might easily have been put to uses far less congruous with its original dedication than those of a hospital for the relief of human suffering.

### THE CHELSEA HOSPITAL FOR WOMEN.

At the beginning of 1919 the wards occupied by wounded officers in 1918 and part of 1917 reverted to normal use, and the number of women patients recorded last year, 760, was therefore double that of the previous twelve months. Nearly all these patients required operative treatment, the large majority being severe and critical cases. During the whole year there were only ten deaths, which gives the extraordinarily low rate of mortality of a little less than 1⅓ per cent. The precautions taken in operating-theatres nowadays against septic influences are not only very minute but also very costly. But when results such as the above are realised, trouble and cost receive full justification. The benefit accruing is not confined to the actual patients but, through the experience gained by the surgeons, it extends to every sufferer who has to undergo operation. The normal income of the Hospital needs the addition of £2,000 to meet present expenditure, and contributions, especially annual subscriptions, are earnestly invited. A great work in the restoration to health and strength is carried on at the Convalescent Home, St. Leonards-on-Sea. Contributions to either Institution will be gratefully received by the Hon. Treasurer or the Secretary at the Hospital, Arthur Street, S.W. 3.

### A DUBLIN SURGEON ON ITS HOSPITALS.

In connection with a mooted centralisation scheme for the many hospitals of the Irish capital, Sir Thomas Myles stated in a recent interview, appearing in the *Irish Independent*, that many of those in favour of the proposal are attracted by the work they see done in large continental or American towns, where there are only one or two great hospitals. His belief is that under such a system one or two men attain a high degree of skill by their specialisation or their extensive experience, but that their services are not available for the great mass of the community. He further considers that the advantages of competition are lost. The rivalry of the hospitals in Dublin means high-class teaching work, as each institution is out to attract students to its school and practice. No doubt one great reason for the suggested consolidation scheme is the financial one. Personally, he objects to the National Insurance Act because the British public get nothing out of it, especially the great middle class. Moreover, the hospitals come off badly, and their visiting staffs are exploited, because serious medical and surgical cases are shunted on to them by the panel practitioners. Unless the authorities behind the Act are going to pay hospitals for the treatment of these patients the financial question will be a serious one. Further, a very large capital sum will be necessary for building the two projected large hospitals, one for the north of the city and the other for the south. Each will need to accommodate at least 800 patients, and on this base he calculated a fund, for building and equipping, of £320,000 to be necessary. The amalgamation of the staffs (which at one time promised great difficulties in the case of London special hospitals), he thinks, will not be hard. One of Sir Thomas's best arguments is clearly his penultimate one. At a time when builders' tenders are such as to frighten a millionare, it is almost impossible to plan the erection of great buildings. Old hospitals, moreover, are not very saleable things, and sites, however eligible, cannot cover the fabulous and mounting cost of bricks and mortar.

### BRADFORD'S MUNICIPAL HOSPITAL.

The Bradford Board of Guardians has finally decided to lease to the Bradford City Council, which has approved the scheme, the whole of the Horton Lane Institution, known as St. Luke's Hospital; the consent of the Ministry of Health is alone now required. St. Luke's, now returned by the military, possesses 1,200 beds, and when the alterations are complete will accommodate some 200 nurses. St. Luke's will thus become a municipal hospital under the control of the Health Committee. Mr. R. S. Dawson, Chairman of the Guardians, claims that this is the first attempt to co-ordinate hospital administration, and that it sets an example which will be followed by other public bodies. The cases will be classified on medical grounds without class distinction. The annual cost of maintenance is expected to be £100,000.

### SIR W. MILLIGAN'S PROPOSAL.

ATTEMPTS in the Provinces to create local funds on the model of King Edward's Hospital Fund for London have several times been made without success. The abortive schemes of such a fund for the Midlands will be in everyone's memory. A suggestion has been made in Manchester by Sir William Milligan that a fund for the whole of Lancashire should be formed, and that the King's consent should be sought to name it after him. It will be remembered that His Majesty has already given his name to the fund for sailors, and that King Edward's name was similarly identified with one fund; and further that London is unique among districts, just as the Navy and Mercantile Marine is a Service apart. But this does not affect the main point in Sir William Milligan's proposal. The wonder is that a plan appealing to many minds should never have been carried out successfully. Manchester is busy in the attempt to raise money for hospitals. Will its attempt lead to more than an immediate sum? Is there in the present effort any prospect of an endowment fund such as King Edward's Fund has given to London? If not, what are the objections that Sir William seems to fear?

### GUARDIANS AND A MEDICAL SUPERINTENDENT'S RESIDENCE.

THE Edmonton Board of Guardians is considering how best to provide a private residence for their medical superintendent, Dr. Mort. When the matter was considered some years ago no action resulted. The Guardians have now resolved that "nine years having passed since the question of providing a private residence away from the hospital for the medical superintendent was considered, also in consideration of his family, that a committee be appointed with power to add to their number to prepare and submit a scheme to the Board on the subject."

### AN INNOVATION AT AN INFIRMARY.

THE authorities of the Post Certificate School of Midwifery at the Royal Lying-in Hospital, York Road, are anxious that their pupils should have the advantage of the experience in regard to venereal diseases which can be obtained at the Lambeth Infirmary, and the guardians have decided to grant the facilities required. It is suggested that each pupil should attend at the infirmary every day for a week, and assist in the wards. Dr. Lionel Baly, the medical superintendent, pointed out to the guardians that possibly the Ministry of Health would make a grant towards the cost of the special instruction, and the guardians have decided to qualify for any grant made by the State in this direction.

### HOSPITAL NEEDED AT DURBAN, SOUTH AFRICA.

THE Durban and Coastal branch of the British Medical Association has addressed a letter to the Mayor and Town Council of Durban calling their attention to the extremely unsatisfactory accommodation for infectious diseases. The Association has reminded the Council that plans for an epidemic hospital in Durban were laid before (and approved by) the Association eight years ago, and that the Association strongly desired that building should proceed at once. No hospital has yet been built. Accommodation for infectious diseases should be provided at once whilst awaiting the erection of a permanent building. Durban is a favourite winter health resort and Durban medical men want it to be made clear that they, as a profession, are not to blame for the lack of the hospital needed.

### THE RESIGNATIONS AT NEWCASTLE.

THE Governors of the Royal Victoria Infirmary, Newcastle, having confirmed the change in Statute 17, whereby the house committee is deprived of the last word in fixing the salaries of the officials, lose by resignation the services of five or possibly six members. As the committee pointed out, the effect of the change is to deprive the executive committee of part of its powers, and is one more attempt by the general body to make the transaction of business impossible. So-called democratic control at Newcastle has already led to inflated committees, and the situation threatens to become impossible unless or until the governors realise the necessity of a division of responsibility during the term of office of its chosen executive.

### THIS WEEK'S DRUG MARKET.

THE market shows signs of an early revival of activity and we must not be surprised if the upward tendency of prices begins afresh. The greater proportion of the inquiry comes from overseas; since the Armistice home requirements have had first attention—which is as it should be—and hospital dispensaries as well as chemists' shops are now fairly well stocked. If the shelves are not full there is enough of most things to go on with for the time being. It is now the turn of the rest of the world, and this is a large business and likely to keep the market active for many months to come. Until the demands from overseas are supplied from the limited stocks and slowly widening outlets of supply prices are likely to be at least maintained, and we see no reason to expect a break in values.

